# Asymptotic analysis of in-plane dynamic problems for elastic media with rigid clusters of small inclusions

**DOI:** 10.1098/rsta.2021.0392

**Published:** 2022-11-28

**Authors:** Michael J. Nieves, Alexander B. Movchan

**Affiliations:** ^1^ School of Computer Science and Mathematics, Keele University,Keele ST5 5BG, UK; ^2^ Department of Mathematical Sciences, University of Liverpool, Liverpool L69 7ZL, UK

**Keywords:** in-plane elastodynamics, clusters of inclusions, Dirichlet problems, asymptotic analysis, meso-scale approximations

## Abstract

We present formal asymptotic approximations of fields representing the in-plane dynamic response of elastic solids containing clusters of closely interacting small rigid inclusions. For finite densely perforated bodies, the asymptotic scheme is developed to approximate the eigenfrequencies and the associated eigenmodes of the elastic medium with clamped boundaries. The asymptotic algorithm is also adapted to address the scattering of in-plane waves in infinite elastic media containing dense clusters. The results are accompanied by numerical simulations that illustrate the accuracy of the asymptotic approach.

This article is part of the theme issue ‘Wave generation and transmission in multi-scale complex media and structured metamaterials (part 2)’.

## 1. Introduction

Vibration is a phenomenon that can promote a range of undesirable effects in engineering structures and materials, compromising their durability. Understanding this is a crucial in the industrial application of materials that may contain flaws or have a rationally designed microstructure formed from inhomogeneities, as found in composite materials. When these materials possess numerous closely situated small impurities, the associated stress concentrations can be amplified, further compromising the material integrity when vibrations propagate through them. Thus, methods for quickly and efficiently establishing the dynamic response of such media in design processes are of vital importance.

Here, we aim to develop an asymptotic approach to describe in-plane waves propagating in elastic materials that contain clusters of closely interacting small impurities having various shapes. In particular, our focus is to illustrate how the approach is effective in capturing the cluster’s influence on vibration processes, including (i) the scattering of waves in infinite elastic media and (ii) vibration modes for finite elastic media, when the shape and size of inclusions become important.

Analytical methods that aid in identifying how large collections of defects or obstacles scatter waves in a medium have been the subject of intensive research effort. The classical work of Foldy [1], appearing in the middle of the twentieth century, led to the description of effective wavenumbers and wavefields propagating through random arrays of N sound-soft isotropic scatterers. Lax [2] later developed this theory to handle anisotropic and inelastic scatterers. The theory provides an effective description of the scattering properties of media with dense microstructures not characterizable without, for instance, an extensive use of microscopy imaging techniques. Corrections to the effective wavenumbers in [1,2] for sparse random arrays of scatterers have been justified in [3]. Effective wavenumbers for elastic wavefields interacting with random arrays of cylinders were tackled with the T-matrix method in [4,5] and for inclusions in plates with the multipole method [6]. Multiple waves have been shown to be supported by random particulates in [7,8]. A comprehensive overview of mathematical approaches developed for treating scattering problems is presented in the monograph [9].

On the other hand, vibration is a fundamental component in the non-destructive evaluation of materials. In connection with this, special attention has been given to solving inverse problems in scattering theory to characterize defects in elastic materials, including clusters of point scatterers [10,11], finite-sized obstacles [12,13] and inclusions with unknown surface conditions in [14].

Analytical procedures for modelling the influence of inclusions on dynamic properties of materials are also useful in designing composites for practical applications. Amenable to a range of approaches such as Floquet–Bloch theory, transfer matrix methods and hybrid approaches involving multiple scattering techniques and the Wiener–Hopf method [15], the associated models also shed light on possible designs of new devices with exotic properties including energy amplification [16], wave guiding [17–19], shielding [20,21], neutrality [22], localization [23] and cloaking [24,25]. Further, in this direction, at specific frequencies approximate theories such as homogenization [26,27] can be applied to capture the effective response of composites with periodic or statistically determined microstructures [28].

For finite media, one seeks eigenvalues and eigenfunctions of partial differential operators to determine how a material vibrates. The method of compound asymptotic approximations, developed in [29,30], provides highly accurate approximations of these dynamic quantities for the Laplacian for various boundary value problems in media with dilute arrangements of small impurities [31–35]. We refer to [36–39] for alternative asymptotic expansions for eigenvalues and eigenfunctions of the Laplacian in domains with a single cavity and [40] for membranes with impedance-type inclusions. Boundary layer approaches have been used in [41] to provide complete asymptotic expansions of eigenfrequencies for a three-dimensional elastic medium with a small void.

Compound asymptotic approximations [29,30] require the appropriate combination of model problems that describe (i) infinite domains with an individual inclusion and (ii) problems in the finite domain without inclusions. The approximations are accurate up to and including the boundaries of the medium and are efficient in the low-frequency regime, where the frequency of vibration does not compete with the defect size. Configurations with several moderately close holes have also been analysed with the so-called functional analytic approach [42] in [43,44].

The method of meso-scale asymptotic approximations [45] was developed in [46,47] to handle problems for media with large clusters of closely interacting small impurities. The approximations require the computation of appropriate weights of model solutions. The weights solve an inhomogeneous algebraic system representing the leading errors induced in the boundary conditions by the proposed asymptotic scheme. This system captures the interaction of the inclusions, taking into account their separation and the individual size and shape of the inclusion. Thus, large data sets obtained via scans or micrographs, which can be executed quickly owing to recent technological advances, are easily used by meso-scale approximations in capturing a range of microscale phenomena. This approach also becomes important in, for instance, determining stress concentrations near particular inclusions within a composite that could otherwise be missed in homogenization models.

Meso-scale approximations have also been shown to be effective in modelling the response of elastic solids with rigid and free clouds of impurities [48,49], low frequency acoustic problems involving rigid defect clusters [50] and steady-state heat conduction in densely packed composites [51]. The approximations have also proven to be useful in modelling flows involving fluids interacting with many small obstacles within narrow spaces [52], important for understanding ${\text{CO}}_{2}$-sequestration processes.

More recently, meso-scale approximations have been produced to model vibration in both finite or infinite membranes containing clusters of rigid movable masses distributed along a contour [53]. There, the scheme developed uses functions acting as solutions to suitable model problems of the Helmholtz operator, moving away from the low-frequency boundary layers previously applied in [29,30,50]. In fact, this provides a way to address modes at higher frequencies, which we demonstrate here in modifying the approach of [53] to develop asymptotic models for in-plane elastodynamic problems for densely perforated media.

In §2, we describe the problem for in-plane vibration modes of a finite elastic medium containing many inclusions. Sections 3 and 4 are dedicated to developing an approximation for eigenfrequencies and eigenfunctions of the elastic body and serve to introduce the model problems used and to develop the associated asymptotic algorithm, respectively. Numerical simulations demonstrating the efficiency of the asymptotic approach of §4 are then given in §5 for elastic solids containing a range of configurations of perforations. These illustrations use Green’s tensor for the interior of the disc, presented in electronic supplementary material, appendix A. Further examples of the effectiveness of the approach are also given in electronic supplementary material, appendix B. In §6, we show that the boundary layers of §3 can be adapted to approximate the scattering response produced by a cluster of inclusions in an infinite planar elastic body. This section also contains a homogenization approximation for the cluster that is derived in electronic supplementary material, appendix C. Accompanying §6 is also a numerical simulation demonstrating the accuracy of the approach when compared with the finite element method. Data for this numerical example is given in electronic supplementary material, appendix D. In §7, we provide some conclusions and future perspectives on the work presented.

## 2. Formulation of the problem

**Figure 1.**
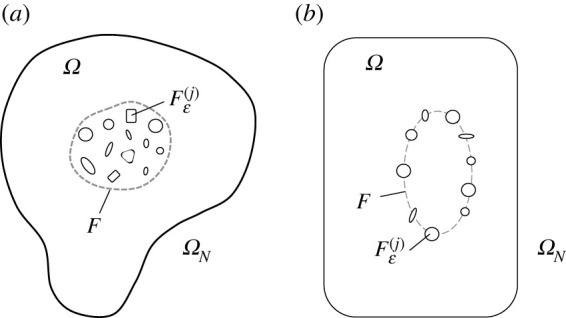
Examples of $\Omega_{N}$. (*a*) The inclusions, having smooth boundaries, are distributed inside a subdomain F of Ω. (*b*) Inclusions positioned along a contour F in the domain Ω.

We consider a two-dimensional elastic material occupying the set Ω, assumed to have a smooth boundary. Contained inside this elastic medium are small rigid obstacles $F_{\varepsilon }^{\left(j\right)}$, j=1,…,N. Here, N is large and ε is a small parameter governing the nominal size of these inclusions. Each obstacle $F_{\varepsilon }^{\left(j\right)}$ has a centre ${\text{O}}^{\left(j\right)}$, 1≤j≤N. The obstacles either occupy a domain or are located on a contour F contained inside Ω and separated by a finite distance from ∂Ω (figure 1). The minimum separation between the centres of two neighbouring obstacles in F is denoted by d, where $d^{n}N=O(1)$ where n=1 for a contour F and n=2 for a domain F. We are concerned with the dynamic response of the elastic medium in $\Omega_{N}:=\Omega \setminus \cup_{j=1}^{N}F_{\varepsilon }^{\left(j\right)}$. Some example configurations of the domains $\Omega_{N}$, considered here, are shown in figure 1.

The eigenmodes of $\Omega_{N}$ are solutions of

2.1 $$\begin{array}{rl} & \mu \Delta {\text{U}}_{N}(\text{x},\omega_{N})+(\lambda +\mu )\nabla (\nabla \cdot {\text{U}}_{N}(\text{x},\omega_{N}))+\rho \omega_{N}^{2}{\text{U}}_{N}(\text{x},\omega_{N})=\text{0},\;\text{x}\in \Omega_{N}, \end{array}$$

2.2 $$\begin{array}{rl} & {\text{U}}_{N}(\text{x},\omega_{N})=\text{0},\;\text{x}\in \partial \Omega \end{array}$$

2.3 $$\begin{array}{rl} \mathrm{and}\; & {\text{U}}_{N}(\text{x},\omega_{N})=\text{0},\;\text{x}\in \partial F_{\varepsilon }^{\left(j\right)},\;1\leq j\leq N, \end{array}$$

with $\omega_{N}$ being the eigenfrequency of the medium and ${\text{U}}_{N}(\text{x},\omega_{N})$ the corresponding mode. Additionally, ρ is the material density and λ and μ are the Lamé parameters. Here, components of the eigenmodes represent complex displacements. First we will approximate the eigenfrequencies and the associated modes. Their direct applications include eigenfunction expansions for solutions to transient problems and Green’s tensors in sufficiently low-frequency regimes.

## 3. Model problems

To construct approximate representations of the eigenmodes (see (2.1)–(2.3)), we will use model problems associated with Ω and the small inclusions $\omega_{\varepsilon }^{\left(j\right)}$, j=1,…,N.

### (a) Singular solution for the infinite body subjected to point forces

Let $\text{x},\text{y}\in R^{2}$ and Γ(x,y,ω) be a 2×2 matrix satisfying

$$\mu \Delta_{\text{x}}\Gamma (\text{x},\text{y},\omega )+(\lambda +\mu )\nabla_{\text{x}}(\nabla_{\text{x}}\cdot \Gamma (\text{x},\text{y},\omega ))+\rho \omega^{2}\Gamma (\text{x},\text{y},\omega )+\delta (\text{x}-\text{y})\text{I}=\text{O},$$

where I is the 2×2 identity matrix and O is the 2×2 zero matrix. The jth column of this matrix corresponds to the response of an infinite body subjected to an oscillating point force aligned with the $x_{j}$-axis, j=1,2, at y of unit amplitude and radian frequency ω. The matrix Γ has the form

3.1 $$\Gamma (\text{x},\text{y},\omega )=\frac{1}{\mu }[\psi (|\text{x}-\text{y}|)\text{I}+\chi (|\text{x}-\text{y}|)\frac{\left(\text{x}-\text{y})⊗(\text{x}-\text{y}\right)}{|\text{x}-\text{y}{|}^{2}}],$$

where $k_{s}=\sqrt{\rho \omega_{N}^{2}/\mu }$, $k_{p}=\sqrt{\rho \omega_{N}^{2}/\left(\lambda +2\mu \right)}$, represent the wavenumbers for shear and pressure waves, respectively,

$$\psi (r)=\frac{i}{4}\left(H_{0}^{\left(1\right)}(k_{s}r)-\left\{\frac{H_{1}^{\left(1\right)}(k_{s}r)}{k_{s}r}-{\left(\frac{k_{p}}{k_{s}}\right)}^{2}\frac{H_{1}^{\left(1\right)}(k_{p}r)}{k_{p}r}\right\}\right)$$

and

$$\chi (r)=\frac{i}{4}\left(H_{2}^{\left(1\right)}(k_{s}r)-{\left(\frac{k_{p}}{k_{s}}\right)}^{2}H_{2}^{\left(1\right)}(k_{p}r)\right).$$

### (b) Dirichlet problem for an inclusion in the infinite planar body

By ${\text{U}}_{\varepsilon }^{\left(j\right)}(\text{x},\omega )$ we denote another 2×2 matrix satisfying the following elastodynamic Dirichlet problem

$$\mu \Delta_{\text{x}}{\text{U}}_{\varepsilon }^{\left(j\right)}(\text{x},\omega )+(\lambda +\mu )\nabla_{\text{x}}(\nabla_{\text{x}}\cdot {\text{U}}_{\varepsilon }^{\left(j\right)}(\text{x},\omega ))+\rho \omega^{2}{\text{U}}_{\varepsilon }^{\left(j\right)}(\text{x},\omega )=\text{O},$$

where $\text{x}\in R^{2}\setminus F_{\varepsilon }^{\left(j\right)}$, and

3.2 $${\text{U}}_{\varepsilon }^{\left(j\right)}(\text{x},\omega )=\text{I},\;\text{x}\in \partial F_{\varepsilon }^{\left(j\right)}.$$

In addition, at infinity ${\text{U}}_{\varepsilon }^{\left(j\right)}$ has the asymptotic representation

3.3 $${\text{U}}_{\varepsilon }^{\left(j\right)}∼{\text{U}}_{\varepsilon }^{\left(s,j\right)}(k_{s}|\text{x}|)+{\text{U}}_{\varepsilon }^{\left(p,j\right)}(k_{p}|\text{x}|),$$

where the vector functions ${\text{U}}_{\varepsilon }^{\left(s,j\right)}(k_{s}|\text{x}|)$ and ${\text{U}}_{\varepsilon }^{\left(p,j\right)}(k_{p}|\text{x}|)$, corresponding to the far-field shear and longitudinal motions of the medium, respectively, satisfy the Sommerfeld radiation conditions

3.4 $$\frac{\partial }{\partial |\text{x}|}{\text{U}}_{\varepsilon }^{\left(m,j\right)}(k_{m}|\text{x}|)-ik_{m}{\text{U}}_{\varepsilon }^{\left(m,j\right)}(k_{m}|\text{x}|)=O\left(\frac{1}{|\text{x}{|}^{3/2}}\right),\;m=s,p.$$

The first and second columns of ${\text{U}}_{\varepsilon }^{\left(j\right)}(\text{x},\omega )$ give the wave field produced by harmonically displacing the inclusion in the $x_{1}$- and $x_{2}$-directions, respectively. For the circular inclusion, with centre ${\text{O}}^{\left(j\right)}$ and radius $r_{\varepsilon }^{\left(j\right)}$, the matrix function ${\text{U}}_{\varepsilon }^{\left(j\right)}(\text{x},\omega )$ takes the form

3.5 $${\text{U}}_{\varepsilon }^{\left(j\right)}(\text{x},\omega )=c_{\varepsilon }^{\left(j,1\right)}\Gamma (\text{x},{\text{O}}^{\left(j\right)},\omega )+c_{\varepsilon }^{\left(j,2\right)}\Delta_{\text{x}}\Gamma (\text{x},{\text{O}}^{\left(j\right)},\omega )$$

with the constants $c_{\varepsilon }^{\left(j,m\right)}$, m=1,2, incorporating information about the boundary conditions and the inclusion size, being given by

$$c_{\varepsilon }^{\left(j,1\right)}=k_{s}(k_{s}c_{\varepsilon }^{\left(j,2\right)}+8\mu B_{\varepsilon }^{\left(j\right)})\;\mathrm{and}\;c_{\varepsilon }^{\left(j,2\right)}=\frac{8\mu k_{s}B_{\varepsilon }^{\left(j\right)}}{k_{s}^{2}-k_{p}^{2}}\left\{{\left(\frac{k_{s}}{k_{p}}\right)}^{2}\frac{H_{2}^{\left(1\right)}(k_{s}r_{\varepsilon }^{\left(j\right)})}{H_{2}^{\left(1\right)}(k_{p}r_{\varepsilon }^{\left(j\right)})}-1\right\},$$

and

$$B_{\varepsilon }^{\left(j\right)}=-\frac{iH_{2}^{\left(1\right)}(k_{p}r_{\varepsilon }^{\left(j\right)})}{k_{s}\{H_{2}^{\left(1\right)}(k_{s}r_{\varepsilon }^{\left(j\right)})H_{0}^{\left(1\right)}(k_{p}r_{\varepsilon }^{\left(j\right)})+H_{0}^{\left(1\right)}(k_{s}r_{\varepsilon }^{\left(j\right)})H_{2}^{\left(1\right)}(k_{p}r_{\varepsilon }^{\left(j\right)})\}}.$$

### (c) Green’s tensor for the finite planar body without holes

The last model field we require is Green’s tensor G(x,y,ω) for the planar body Ω, which solves the boundary value problem

$$\mu \Delta_{\text{x}}\text{G}(\text{x},\text{y},\omega )+(\lambda +\mu )\nabla_{\text{x}}(\nabla_{\text{x}}\cdot \text{G}(\text{x},\text{y},\omega ))+\rho \omega^{2}\text{G}(\text{x},\text{y},\omega )+\delta (\text{x}-\text{y})\text{I}=\text{O},$$

for x,y∈Ω and

3.6 G(x,y,ω)=O,x∈∂Ω, y∈Ω.

The regular part R(x,y,ω) of Green’s tensor is then defined as R(x,y,ω)=Γ(x,y,ω)−G(x,y,ω). The components of the matrix function R can be computed easily for simple geometries. If Ω is a circular plate, Helmholtz’s decomposition and Graf’s addition theorem can be applied to compute the matrix R. The corresponding solution is given in electronic supplementary material, appendix A.

## 4. Formal approximation of eigenfrequencies and eigenmodes

### (a) Approximation of the eigenfunction

We develop the asymptotic algorithm for the case when the small rigid inclusions are circular. We note a similar algorithm can be carried out for rigid inclusions with arbitrary shapes.

For circular inclusions, the approximation to the complex vector function ${\text{U}}_{N}$ is sought as

4.1 $${\text{U}}_{N}(\text{x},\omega_{N})=\sum_{j=1}^{N}{\text{W}}_{\varepsilon }^{\left(j\right)}(\text{x},\omega_{0})\beta^{\left(j\right)}+r_{N}(\text{x})$$

with

4.2 $${\text{W}}_{\varepsilon }^{\left(j\right)}(\text{x},\omega_{0})={\text{U}}_{\varepsilon }^{\left(j\right)}(\text{x},\omega_{0})-c_{\varepsilon }^{\left(j,1\right)}\text{R}(\text{x},{\text{O}}^{\left(j\right)},\omega_{0})-c_{\varepsilon }^{\left(j,2\right)}\Delta_{\text{y}}\text{R}(\text{x},{\text{O}}^{\left(j\right)},\omega_{0}),$$

and $\beta^{\left(j\right)}$ are constant vectors to be determined. The matrix functions ${\text{W}}^{\left(j\right)}$ are constructed in such a way that they satisfy

$$\mu \Delta {\text{W}}_{\varepsilon }^{\left(j\right)}(\text{x},\omega_{0})+(\lambda +\mu )\nabla (\nabla \cdot {\text{W}}_{\varepsilon }^{\left(j\right)}(\text{x},\omega_{0}))+\rho \omega_{0}^{2}{\text{W}}^{\left(j\right)}(\text{x},\omega_{0})=\text{0},$$

for $\text{x}\in \Omega \setminus F_{\varepsilon }^{\left(j\right)}$, 1≤j≤N. Additionally, the terms that use the regular part of Green’s tensor in Ω in (4.2) ensure the trace of ${\text{W}}_{\varepsilon }^{\left(j\right)}$, 1≤j≤N, on ∂Ω is zero (see (3.5) and (3.6)). For inclusions with general shapes, these terms are based on the individual representations of ${\text{U}}_{\varepsilon }^{\left(j\right)}$, 1≤j≤N, at infinity and are constructed to ensure a small error is produced on ∂Ω by ${\text{W}}_{\varepsilon }^{\left(j\right)}$, 1≤j≤N.

We note that on $\partial F_{\varepsilon }^{\left(j\right)}$, ${\text{W}}_{\varepsilon }^{\left(j\right)}$ is non-zero for j=1,…,N, and therefore their presence in (4.1) seemingly creates a difficulty in satisfying the boundary conditions (2.3) on the interior boundaries. However, we have freedom to choose the vector coefficients $\beta^{\left(j\right)}$, 1≤j≤N, in (4.1) that later enables these conditions to be satisfied to a high order of accuracy.

Hence, it follows from the above, §3 and (4.1) that

$$\mu \Delta r_{N}(\text{x})+(\lambda +\mu )\nabla (\nabla \cdot r_{N}(\text{x}))+\rho \omega_{N}^{2}r_{N}(\text{x})=\rho (\omega_{0}^{2}-\omega_{N}^{2})\sum_{j=1}^{N}{\text{W}}_{\varepsilon }^{\left(j\right)}(\text{x},\omega_{0})\beta^{\left(j\right)},$$

for $\text{x}\in \Omega_{N}$ and $r_{N}(\text{x})=\text{0}$ for x∈∂Ω. Further, (2.3) and (3.2) together with (4.1) imply that for $\text{x}\in \partial F_{\varepsilon }^{\left(k\right)}$, 1≤j≤N, the remainder satisfies

$$r_{N}(\text{x})=-S^{\left(k\right)}(\text{x},{\text{O}}^{\left(k\right)},\omega_{0})\beta^{\left(k\right)}-\sum_{\begin{array}{c} 1\leq j\leq N\\ j\neq k \end{array}}T^{\left(j\right)}(\text{x},{\text{O}}^{\left(j\right)},\omega_{0})\beta^{\left(j\right)},$$

with

$$S^{\left(k\right)}(\text{x},\text{y},\omega_{0})=\text{I}-c_{\varepsilon }^{\left(k,1\right)}\text{R}(\text{x},{\text{O}}^{\left(k\right)},\omega_{0})-c_{\varepsilon }^{\left(k,2\right)}\Delta_{\text{y}}\text{R}(\text{x},{\text{O}}^{\left(k\right)},\omega_{0})$$

and

$$T^{\left(j\right)}(\text{x},\text{y},\omega_{0})=c_{\varepsilon }^{\left(j,1\right)}\text{G}(\text{x},{\text{O}}^{\left(j\right)},\omega_{0})+c_{\varepsilon }^{\left(j,2\right)}\Delta_{\text{y}}\text{G}(\text{x},{\text{O}}^{\left(j\right)},\omega_{0}).$$

Next, the Taylor expansion about $\text{x}={\text{O}}^{\left(k\right)}$ gives for $\text{x}\in \partial F_{\varepsilon }^{\left(k\right)}$, 1≤k≤N,

$$\begin{array}{rl} r_{N}(\text{x}) & \;∼-S^{\left(k\right)}({\text{O}}^{\left(k\right)},{\text{O}}^{\left(k\right)},\omega_{0})\beta^{\left(k\right)}-\sum_{\begin{array}{c} 1\leq j\leq N\\ j\neq k \end{array}}T^{\left(j\right)}({\text{O}}^{\left(k\right)},{\text{O}}^{\left(j\right)},\omega_{0})\beta^{\left(j\right)}. \end{array}$$

The last conditions provide an opportunity to obtain the unknown vector coefficients $\beta^{\left(j\right)}$, 1≤j≤N, and to remove the leading-order discrepancy in the right-hand side. Thus, setting

4.3 $$\text{0}=S^{\left(k\right)}({\text{O}}^{\left(k\right)},{\text{O}}^{\left(k\right)},\omega_{0})\beta^{\left(k\right)}+\sum_{\begin{array}{c} 1\leq j\leq N\\ j\neq k \end{array}}T^{\left(j\right)}({\text{O}}^{\left(k\right)},{\text{O}}^{\left(j\right)},\omega_{0})\beta^{\left(j\right)},$$

for 1≤k≤N, provides a homogeneous system for the required coefficients.

### (b) Approximation of eigenfrequencies

The degeneracies of the system (4.3) allow for the leading-order approximation $\omega_{0}$ to $\omega_{N}$ to be determined, while simultaneously ensuring the sought coefficients $\beta^{\left(j\right)}$, 1≤j≤N, are non-trivial. With this in mind, (4.3) can be written in the matrix form

4.4 $$D(\omega_{0})\beta =\text{0}\;\text{with }D(\omega_{0})={\text{I}}_{2N}+G(\omega_{0}).$$

Here, 0 represents the 2N-dimensional zero vector and ${\text{I}}_{2N}$ is the 2N×2N identity matrix. In addition, $G(\omega_{0})={\left[G_{ij}(\omega_{0})\right]}_{i,j=1}^{N}$ is a 2N×2N matrix with matrix entries

$$G_{ij}(\omega_{0})=\left\{ \begin{array}{ll} -c_{\varepsilon }^{\left(i,1\right)}\text{R}({\text{O}}^{\left(i\right)},{\text{O}}^{\left(i\right)},\omega_{0})-c_{\varepsilon }^{\left(i,2\right)}\Delta_{\text{y}}\text{R}({\text{O}}^{\left(i\right)},{\text{O}}^{\left(i\right)},\omega_{0}), & \text{for }i=j\\ c_{\varepsilon }^{\left(j,1\right)}\text{G}({\text{O}}^{\left(i\right)},{\text{O}}^{\left(j\right)},\omega_{0})+c_{\varepsilon }^{\left(j,2\right)}\Delta_{\text{y}}\text{G}({\text{O}}^{\left(i\right)},{\text{O}}^{\left(j\right)},\omega_{0}), & \text{otherwise}, \end{array}\right.$$

for 1≤i,j≤N. Additionally, $\beta ={\left({\left(\beta^{\left(1\right)}\right)}^{T},\ldots ,{\left(\beta^{\left(N\right)}\right)}^{T}\right)}^{T}$ is the non-trivial solution of system (4.4). Note in the case of a single inclusion, i.e. N=1, the required matrix $D(\omega_{0})$ is simply

4.5 $$D(\omega_{0})=\text{I}-c_{\varepsilon }^{\left(1,1\right)}\text{R}({\text{O}}^{\left(1\right)},{\text{O}}^{\left(1\right)},\omega_{0})-c_{\varepsilon }^{\left(1,2\right)}\Delta_{\text{y}}\text{R}({\text{O}}^{\left(1\right)},{\text{O}}^{\left(1\right)},\omega_{0})$$

following directly from the above asymptotic algorithm with obvious modifications. The non-trivial vector coefficients found in (4.4) are then obtained from the roots $\omega_{0}$ of $\det(D(\omega_{0}))=0$.

## 5. Numerical illustrations for finite elastic media

Here, we demonstrate numerically the accuracy of the method developed by determining eigenfrequencies and eigenmodes for elastic bodies with circular inclusions. We begin with the case of a single inclusion to illustrate the main ideas before tackling clusters in elastic media. Below, Ω is the disc of radius R=5 m and centre at the origin and occupied by an elastic material characterized by the Lamé constants $\lambda =\mu =1\;{\mathrm{Nm}}^{-2}$ and density $\rho =1\;\mathrm{kg}\;m^{-3}$. The boundary layers for Ω required by the asymptotic approximation are easily retrievable (see electronic supplementary material, appendix A).

### (a) A disc containing a single inclusion

#### (i) Prediction of eigenfrequencies

**Figure 2.**
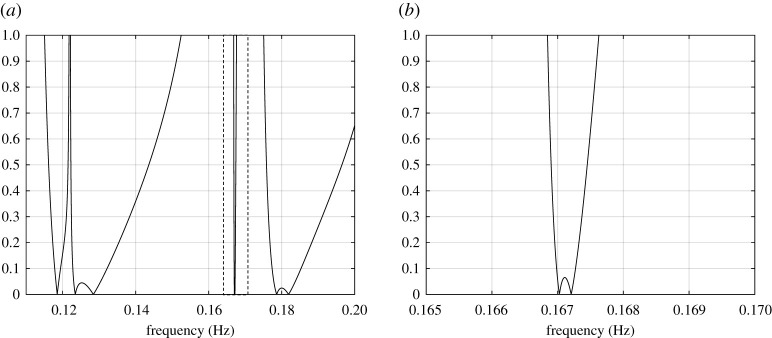
Behaviour of $|\text{det}(D(\omega_{0}))|$ as a function of the frequency of vibration. (*a*) The plot of $|\text{det}(D(\omega_{0}))|$ for 0.1≤f≤0.2. (*b*) Magnification of the plot in (*a*) within the dashed box. Eigenfrequencies are given by the zeros of $|\text{det}(D(\omega_{0}))|$ shown in table 1.

Here Ω contains a small circular hole of radius 0.1 m, with centre (0.5 m, 0.5 m). Approximations $\omega_{0}$ of the eigenfrequencies of this elastic body are traceable from the roots of $\det(D(\omega_{0}))=0$, which uses (4.5). In figure 2*a*, $|\det(D(\omega_{0}))|$ is shown as a function of f, the frequency of vibration in Hertz ($f=\omega_{0}/2\pi$), within the frequency range 0.1≤f≤0.2. There, degeneracies of $D(\omega_{0})$ occur for seven values of the frequency parameter f. In particular, figure 2*b* indicates that the eigenfrequencies of the system can appear very close to each other.

**Table 1.** The first 10 eigenfrequencies (f in Hz) for a disc of radius 5 m and centre (0 m, 0 m) containing a small circular inclusion with radius 0.1 m and centre (0.5 m, 0.5 m).

mode	f from (4.4)	COMSOL	relative error	mode	f from (4.4)	COMSOL	relative error
1	0.11852	0.1186	0.064%	6	0.17863	0.17869	0.039%
2	0.12348	0.12348	0.007%	7	0.18198	0.18185	0.019%
3	0.12842	0.12847	0.039%	8	0.21237	0.21259	0.105%
4	0.16702	0.16725	0.134%	9	0.21544	0.21551	0.034%
5	0.16721	0.16743	0.134%	10	0.21556	0.21562	0.028%

**Figure 3.**
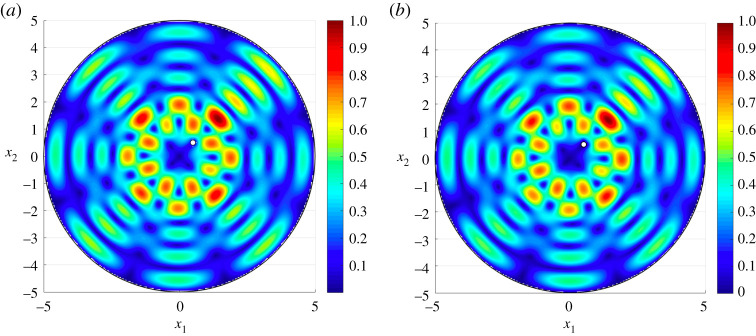
The total displacement for the 105th eigenmode corresponding to the eigenfrequency f=0.61169 Hz given by (*a*) the asymptotic approximation (4.1) and (*b*) COMSOL MultiPhysics. The finite element solution, involving the computation of 112 288 d.f., was computed on a mesh with 27 898 triangular elements, 348 edge elements and eight vertex elements. The average absolute error between (*a*,*b*) is $2.04\times 10^{-2}$. (Online version in colour.)

The values of the first 10 eigenfrequencies for the considered system, based on the solution of $\det(D(\omega_{0}))=0$ are shown table 1. Accompanying these are the predictions for these eigenfrequencies based on a finite-element analysis of the problem performed in COMSOL Multiphysics 5.3 using the Structural Mechanics module (further details of the computation are found in figure 3). The relative error between the analytical predictions and those based on the finite-element method is also given. Based on this comparative analysis, it is clear this condition allows for an excellent prediction of the eigenfrequencies. In electronic supplementary material, appendix B, we present further computations of modes associated with several eigenfrequencies found in table 1 to illustrate the effectiveness of the method presented here.

#### (ii) High-frequency eigenmode for the disc with a small hole

As an example showing the robustness of the asymptotic scheme for higher frequencies, we present in figure 3*a* the 105th eigenmode of the considered system. This wave mode has three preferential directions for oscillations in the radial direction. Note here, these oscillations are almost comparable to the size of the small hole. Nevertheless, the approximation produces an excellent match with the result of COMSOL in figure 3*b*. Additionally, based on the theory developed here, the prediction for the associated eigenfrequency as 0.61169 Hz, whereas COMSOL predicts this to be 0.61236 Hz.

### (b) Dynamics of composites with clusters of small inclusions

Here we highlight the effectiveness of the asymptotics in predicting the dynamic behaviour of multiscale elastic composites with clusters of small inclusions occupying a domain or located along a contour.

#### (i) Inclusions distributed within a subdomain of an elastic medium

**Figure 4.**
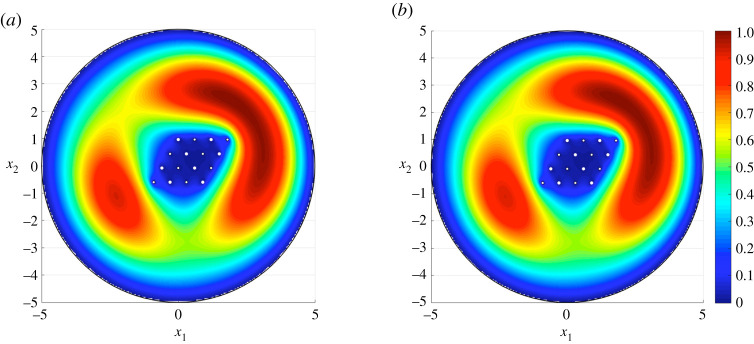
The total displacement associated with the third eigenmode for a disc containing a doubly periodic cluster of 16 circular rigid inclusions arranged in a rhomboid. Here, we present results obtained from (*a*) the asymptotic approximation (4.1) and (*b*) COMSOL MultiPhysics where 260 082 d.f. were solved for on a mesh involving 64 614 triangular elements, 828 edge elements and 68 vertex elements. Average absolute error between (*a*,*b*) is $1.7\times 10^{-3}$. (Online version in colour.)

We consider an elastic disc Ω containing N=16 circular inclusions that form a rhomboidal doubly periodic cluster (figure 4). The inclusions in the rhomboid have the centres given by $0.3\{{\left(-3,-2\right)}^{T}+2p{\text{v}}_{\text{1}}+q{\text{v}}_{\text{2}}\}$, for 0≤p,q≤3, where ${\text{v}}_{1}={\left(1,0\right)}^{T},{\text{v}}_{2}={\left(1,\sqrt{3}\right)}^{T}$ and radii equal to 0.04 m if p+q is even and 0.06 m otherwise. COMSOL predicts this medium has the first eigenfrequency 0.13442 Hz, whereas the asymptotic scheme predicts the value of this eigenfrequency to be 0.1343 Hz. For the sake of brevity, we do not report the associated first eigenmodes here.

Figure 4 shows the asymptotic approach accurately captures the dynamic response of an elastic medium containing a cluster of inclusions at higher eigenfrequencies. The third mode for the medium is presented figure 4*a* according to the asymptotic formulae, which predict the associated eigenfrequency as 0.1584 Hz. In figure 4*b* the computation based on the finite element approach in COMSOL is shown. COMSOL identifies the associated eigenfrequency as 0.15855 Hz. The visual difference between (a) and (b) is again indistinguishable.

#### (ii) Inclusions distributed along a contour in an elastic medium

Here, we consider N=10 rigid inclusions with centres distributed in Ω according to ${\text{O}}^{\left(j\right)}={\left(\text{Re}(z^{\left(j\right)}),\text{Im}(z^{\left(j\right)})\right)}^{T}$, $z^{\left(j\right)}=p^{\left(j\right)}\;e^{-i\pi /4}+i$ with $p^{\left(j\right)}=\cos(2\pi (j-1)/N)+\frac{3}{2}i\sin(2\pi (j-1)/N)$ for j=1,…,N. The inclusion centres represent points located along the ellipse with centre (0 m, 1 m), having semi-major and semi-minor axes 1.5 m and 1 m, respectively, and that is also rotated by π/4 clockwise. Each circular inclusion has the radius 0.05 m. The described medium has a first eigenmode at the eigenfrequency 0.13049 Hz, according to COMSOL, whereas the asymptotic procedure predicts this to be 0.13059 Hz.

**Figure 5.**
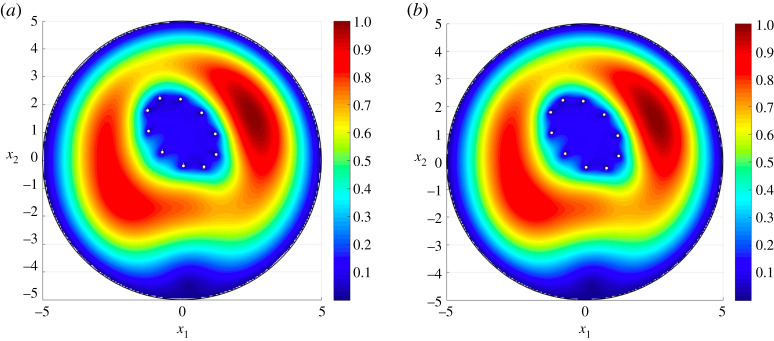
The total displacement associated with the third eigenmode for a disc containing a cluster of 10 inclusions arranged along an elliptical contour. The boundaries of the medium are clamped. Here, we present results obtained from (*a*) the asymptotic approximation (4.1) and (*b*) COMSOL MultiPhysics. The finite-element solution is composed of 221 022 d.f., computed on a mesh with 54 942 triangular elements, 636 edge elements and 44 vertex elements. Average absolute error between (*a*,*b*) is $1.6\times 10^{-3}$. (Online version in colour.)

The third eigenmode for the elastic medium is presented in figure 5. This reveals that the elliptical cluster helps to block the effects of external disturbances within its interior. Similar effects have also been identified for infinite media for membranes and electrostatics [20]. At this frequency, the cluster acts as an elastic analogue to the Faraday cage, which redistributes electrical charge along its surface to suppress the effect of the electrostatic field inside. Once more, the computations related to the asymptotics in (a) provide an excellent agreement with those based on COMSOL, with the eigenfrequency corresponding to this mode being 0.1643 Hz and 0.1641 Hz in figure 5*a*,*b*, respectively. The example discussed above shows the asymptotic scheme is efficient in identifying special dynamic phenomena for finite non-periodic composites, while taking into account the interactions between internal and exterior boundaries.

## 6. Scattering of elastic waves by clusters of inclusions

Next we adapt the tools developed in the previous sections to create asymptotic approximations for fields associated with the scattering of elastic waves by rigid clusters.

### (a) Governing equations for the problem of elastic wave scattering

We look for the approximation to the displacement field $U_{N}(\text{x},\omega )$ that is a solution to the problem

6.1 $$\mu \Delta U_{N}(\text{x},\omega )+(\lambda +\mu )\nabla (\nabla \cdot U_{N}(\text{x},\omega ))+\rho \omega^{2}U_{N}(\text{x},\omega )=\text{f}(\text{x}),\;\text{x}\in R^{2}\setminus \cup_{j=1}^{N}F_{\varepsilon }^{\left(j\right)}$$

and

$$U_{N}(\text{x},\omega )=\text{0},\;\text{x}\in \cup_{j=1}^{N}\partial F_{\varepsilon }^{\left(j\right)},$$

with f representing the body force applied to the elastic medium that generates an incident field with frequency ω on the cluster. In what follows, we approximate the field $U_{N}$ by making use of model fields defined in §3*b*, and the solution to an inhomogeneous algebraic system that appears when satisfying the boundary conditions on small inclusions to a high order of accuracy.

We briefly mention that if the cluster is dense, periodic and the inclusion size is exponentially small compared with their separation, then in the low-frequency regime the approximation developed in the next section yields an effective equation for the cluster F. Indeed, for circular inclusions, let $\varepsilon =\text{exp}(-cd^{-2})$, with d being the separation of individual inclusions and c being a parameter determining the inclusion radius ε when compared with d inside a unit cell of the cluster. Then inside the cloud of rigid inclusions, waves are governed by the equation

6.2 $$\begin{array}{rl} & \mu \Delta U_{\mathrm{eff}}(\text{x},\omega )+(\lambda +\mu )\nabla (\nabla \cdot U_{\mathrm{eff}}(\text{x},\omega ))+(\rho \omega^{2}-\frac{4\pi }{c}\frac{\mu (\lambda +2\mu )}{\lambda +3\mu })U_{\mathrm{eff}}(\text{x},\omega )=\text{f}(\text{x}),\;\text{x}\in F, \end{array}$$

where $U_{\mathrm{eff}}$ is the effective wavefield in F. This result is derived in electronic supplementary material, appendix C.

### (b) Asymptotic algorithm

Note it is standard to represent the total field $U_{N}$ as

6.3 $$U_{N}(\text{x},\omega )=U^{i}(\text{x},\omega )+U_{N}^{s}(\text{x},\omega ),$$

where $U^{i}(\text{x},\omega )$ is the field that is incident on the cluster and produced by the load f. This field is assumed to be known and regular in the vicinity of the cluster. It is a solution of

6.4 $$\mu \Delta U^{i}(\text{x},\omega )+(\lambda +\mu )\nabla (\nabla \cdot U^{i}(\text{x},\omega ))+\rho \omega^{2}U^{i}(\text{x},\omega )=\text{f}(\text{x}),\;\text{x}\in R^{2}.$$

In (6.3), $U_{N}^{s}(\text{x},\omega )$ is the scattered field produced by the interaction of the incident wave and the cluster and it remains to provide the approximation to this vector function. With this in mind, we note the scattered field solves

$$\mu \Delta U_{N}^{s}(\text{x},\omega )+(\lambda +\mu )\nabla (\nabla \cdot U_{N}^{s}(\text{x},\omega ))+\rho \omega^{2}U_{N}^{s}(\text{x},\omega )=\text{0},\;\text{x}\in R^{2}\setminus \cup_{j=1}^{N}F_{\varepsilon }^{\left(j\right)}$$

and

$$U_{N}^{s}(\text{x},\omega )=-U^{i}(\text{x},\omega ),\;\text{x}\in \partial F_{\varepsilon }^{\left(j\right)},\;1\leq j\leq N.$$

Far from the cluster, $U_{N}^{s}(\text{x},\omega )$ satisfies analogous radiation conditions to (3.3) and (3.4), except that here $k_{s}=\sqrt{\rho \omega^{2}/\mu }$ and $k_{p}=\sqrt{\rho \omega^{2}/\left(\lambda +2\mu \right)}$. We look for the scattered field in the form

6.5 $$U_{N}^{s}(\text{x},\omega )=\sum_{j=1}^{N}{\text{U}}_{\varepsilon }^{\left(j\right)}(\text{x},\omega ){\text{C}}^{\left(j\right)}+R_{N}(\text{x}),$$

where ${\text{U}}_{\varepsilon }^{\left(j\right)}$ is defined in §3b and ${\text{C}}^{\left(j\right)}$ are constant vectors now to be determined as solutions of an inhomogeneous system. The remainder $R_{N}$ then is a solution of the equation

$$\mu \Delta R_{N}(\text{x})+(\lambda +\mu )\nabla (\nabla \cdot R_{N}(\text{x}))+\rho \omega^{2}R_{N}(\text{x})=\text{0},\;\text{x}\in R^{2}\setminus \cup_{j=1}^{N}F_{\varepsilon }^{\left(j\right)},$$

and on the boundary of small inclusions, i.e. when $\text{x}\in \partial F_{\varepsilon }^{\left(j\right)}$, 1≤j≤N, we have

$$R_{N}(\text{x})=-U^{i}(\text{x},\omega )-{\text{C}}^{\left(j\right)}-\sum_{\begin{array}{c} k\neq j\\ 1\leq k\leq N \end{array}}{\text{U}}_{\varepsilon }^{\left(k\right)}(\text{x},\omega ){\text{C}}^{\left(k\right)},$$

where (6.5) and (3.2) have been used. We then apply the Taylor expansion about $\text{x}={\text{O}}^{\left(j\right)}$ to obtain:

$$R_{N}(\text{x})∼-U^{i}({\text{O}}^{\left(j\right)},\omega )-{\text{C}}^{\left(j\right)}-\sum_{\begin{array}{c} k\neq j\\ 1\leq k\leq N \end{array}}{\text{U}}_{\varepsilon }^{\left(k\right)}({\text{O}}^{\left(j\right)},\omega ){\text{C}}^{\left(k\right)},\;\text{x}\in \partial F_{\varepsilon }^{\left(j\right)},1\leq j\leq N.$$

Here, the leading-order term in the right-hand side can be removed by appropriately choosing the coefficients ${\text{C}}^{\left(j\right)}$, 1≤j≤N. Hence, for 1≤j≤N, we prescribe that these coefficients satisfy

6.6 $${\text{C}}^{\left(j\right)}+\sum_{\begin{array}{c} k\neq j\\ 1\leq k\leq N \end{array}}{\text{U}}_{\varepsilon }^{\left(k\right)}({\text{O}}^{\left(j\right)},\omega ){\text{C}}^{\left(k\right)}=-U^{i}({\text{O}}^{\left(j\right)},\omega ).$$

### (c) Numerical example: scattering of waves by a circular cluster

As a final illustration of the theory developed in §6b, we consider scattering of in-plane elastic waves due to a cluster of inclusions. The cluster has N=14 small circular defects of varying radius, from 0.08 m to 0.16 m, distributed along a circular contour of radius 2.5 m and centre ${\left(0\;m,0\;m\right)}^{T}$ (see electronic supplementary material, appendix D for the data describing the inclusions). The medium is subjected to a horizontally acting sinusoidal force of unit amplitude and frequency f=1/π Hz, located at $\text{y}={\left(-8,0\right)}^{T}$ outside the cluster. Here, $f=-\delta (\text{x}-\text{y}){\left(1,0\right)}^{T}$ in (6.1) and, mathematically, the field $U_{N}$ represents the first column of Green’s tensor for $R^{2}\setminus \cup_{j=1}^{N}F_{\varepsilon }^{\left(j\right)}$. To employ our asymptotic scheme, we require the incident field $U_{N}^{i}(\text{x},\omega )$ (see (6.4)), which is taken as the first column of the matrix Γ in (3.1). The scattered field $U_{N}^{s}(\text{x},\omega )$ is then given by (6.5), (6.6) together with (3.5).

**Figure 6.**
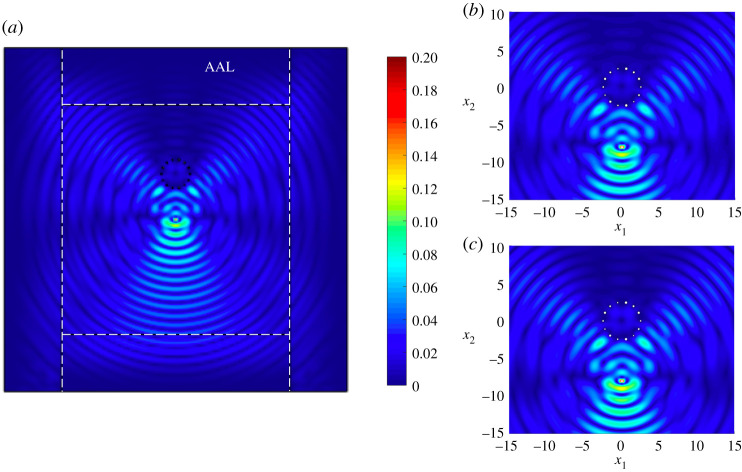
Scattering of waves produced by a non-periodic circular cluster. Total displacement field based on computations in COMSOL is shown in (*a*,*b*) and involve 855 346 d.f. An adaptive absorbing layer (AAL) is included on the exterior of the computational domain, indicated by white dashed lines to reduce reflections. The mesh used to compute the finite-element solution consists of 213 194 triangular elements, 1902 edge elements and 69 vertex elements. Here (*b*,*c*) show a magnification of the results in (*a*) in the neighbourhood of the point source and the circular cluster, with (*c*) based on the asymptotics (6.5) with (6.6). (Online version in colour.)

Figure 6 shows that shear waves produced by the load, whose wavelengths are comparable to the spacing of the inclusions, interact with the cluster and are scattered. Clear preferential directions of the scattered waves can be seen at approximately $45^{\circ }$ and $135^{\circ }$ from the base of the cluster defined relative to the positive $x_{1}$-direction. There are also pressure waves to left and right of the load that interact with the waves reflected by the cluster. As in §5b, the cluster helps to suppress the influence of the external vibrations within its interior. It also produces a shielding effect illustrated by the shadow behind the cluster relative to the load. A magnification of the phenomenon encountered by the source interacting with the cluster is shown in figure 6*b*. The corresponding result based on the asymptotic approximation is shown in figure 6*c*. Once more, we have an excellent agreement between the results in figure 6*b*,*c*.

## 7. Conclusion

Here, we have developed an asymptotic algorithm to model the in-plane dynamic behaviour of both finite and infinite elastic media with clusters of small rigid inclusions. The approach uses boundary layers constructed from functions naturally associated with the partial differential operator embedded in the underlying boundary value problems. As a result, this allows for a greater the range of applicability of the asymptotic results when compared with previous asymptotic approximations [46–51] for quasi-static problems that use boundary layers well suited to static problems. The boundary layers introduced here also take into account the size and shape of small inclusions and, for finite elastic media, account for the influence of the exterior boundary in the considered dynamic processes.

Another key ingredient in constructing the asymptotic approximations are the solutions to certain finite algebraic systems that arise when attempting to satisfy the boundary conditions to a high degree of accuracy. These systems incorporate information about the types of individual inclusions, their size and distribution. The corresponding solutions allow the asymptotic formulae to accurately capture the interaction between the inclusions up to and including the boundaries of the medium. It is important to note that the approximations do not require strict assumptions on the distribution of inclusions within clusters, such as periodicity, or probabilistic conditions on their arrangement that are often easily treated with homogenization techniques.

Further, the asymptotic scheme may provide an efficient alternative to techniques such as the multipole method and T-matrix method used in the analysis of elastic scattering. They require the handling of infinite algebraic systems in capturing the interaction of obstacles that, as mentioned in [9], can lead to numerical problems in their application. In general, the meso-scale technique developed here only requires the multipole expansion of fields for inclusions in isolation, and the subsequent use of these fields in solving finite systems which are easily handled computationally.

The formal asymptotic algorithms presented are also extendable, with modifications, to problems involving defects of different types, e.g. voids, soft inclusions and inertial inclusions such as masses and resonators, and different dynamic problems for elastic media useful in the construction of novel waveguides and structured metamaterials.

Potential applications of the proposed methods include civil engineering, the non-destructive testing of materials and the modal analysis of structures found in, for instance, civil engineering, aerospace and naval architecture where stratified solids are often used. Additionally, the method may open new directions in the design of novel composites that use an embedded microstructure to achieve unconventional macro-level responses for practical purposes.

## Data Availability

Electronic supplementary material is available online at [54].
